# Challenging diagnosis of resistance to thyroid hormone in a patient with COVID-19, pituitary microadenoma and unusual response to octreotide long-acting release test

**DOI:** 10.1530/EDM-23-0146

**Published:** 2024-04-17

**Authors:** Cristian Petolicchio, Sara Brasili, Stefano Gay, Francesco Cocchiara, Irene Campi, Luca Persani, Lara Vera, Diego Ferone, Federico Gatto

**Affiliations:** 1 Endocrinology Unit, IRCCS Ospedale Policlinico San Martino, Genoa, Italy; 2 Endocrinology Unit, Department of Internal Medicine and Medical Specialties (DiMI), University of Genova, Genoa, Italy; 3 Department of Endocrine and Metabolic Diseases, IRCCS Istituto Auxologico Italiano, Milan, Italy

**Keywords:** Adult, Male, White, Italy, Thyroid, Thyroid, Error in diagnosis/pitfalls and caveats, April, 2024

## Abstract

**Summary:**

The resistance to thyroid hormone syndrome (RTHβ) occurs uncommonly and requires a high level of clinical suspicion and specific investigations to reach a precise diagnosis and to avoid unnecessary and potentially harmful therapies. We report a case of a young male patient referred to our unit for SARS-CoV-2 infection and atrial fibrillation with elevated thyroid hormones and non-suppressed thyroid-stimulating hormone (TSH), for which antithyroid therapy was prescribed. A mood disorder was reported in the medical history. The family history was unknown as the patient was adopted. Thyroid-specific antibodies were undetectable, and thyroid ultrasound revealed a normal thyroid gland without nodules. After the resolution of SARS-CoV-2 infection, the diagnostic workup continued, and the pituitary MRI revealed a small area ascribable to a microadenoma. Due to atrial fibrillation, the execution of the T3 test was contraindicated. The octreotide long-acting release (LAR) test showed an initial reduction of free thyroid hormones levels at first administration, which was consistent with the presence of a TSH-secreting pituitary tumour, although an escape from the response was observed after the following two injections of octreotide LAR. Indeed, the genetic investigation revealed a variant in heterozygosity of the THRβ gene (Pro453Ser), thus leading to an RTHβ diagnosis, and, therefore, medical treatment with triiodothyroacetic acid was initiated. After 2 years from the SARS-CoV-2 infection, the patient continues the follow-up at our outpatient clinic, and no other medical interventions are needed.

**Learning points:**

- RTHβ is a rare genetic syndrome characterised by discrepant thyroid function tests and by a dissociation between the observed hormone levels and the expected patient signs and symptoms.
- Features of thyroid hormone deficiency in TR-ß dependent tissues (pituitary gland, hypothalamus, liver and neurosensitive epithelia), as well as thyroid hormone excess in TR-α-dependent tissues (heart, bone, skeletal muscle and brain), may coexist in the same individual.
- Clinical pictures can be different even when the same variant occurs, suggesting that other genetic and/or epigenetic factors may play a role in determining the patient’s phenotype.
- Differentiating RTHβ from a TSH-secreting pituitary tumour is very difficult, especially when a concomitant pituitary adenoma is detected during diagnostic workup. The injection of long-acting somatostatin analogues can help differentiate the two conditions, but it is important to detect any interference in the dosage of thyroid hormones to avoid an incorrect diagnosis.
- Genetic testing is fundamental to prevent unnecessary and potentially harmful therapies.
- Medical treatment with triiodothyroacetic acid was demonstrated to be effective in reducing thyroid hormone excess and controlling symptoms.

## Background

The syndromes of impaired sensitivity to thyroid hormone represent a heterogeneous group of genetic disorders characterised by discrepant thyroid function tests (TFTs) and a dissociation between the observed hormone levels and the expected patient signs and symptoms (1).

The first of these conditions was described by Refetoff in 1967 (2), and it was caused by a homozygous deletion of the THRβ gene, encoding for thyroid hormone receptor beta (TR-β). Instead, most of these disorders are caused by dominant negative heterozygous variants of the gene and are commonly called resistance to thyroid hormone beta (RTHβ) or Refetoff syndrome.

The precise incidence of RTHβ is unknown, but some studies suggest an estimated prevalence between 1:20 000 and 1:40 000 live births with an equal sex preponderance (3).

Family occurrence has been documented in approximately 75% of cases, with typical autosomal dominant inheritance. Since 1989, when *THRß* variants were first identified, over 236 different mutations have been discovered (3).

The *THRβ* is located on chromosome 3p24.2, and pathogenic variants involve three CpG-rich hotspot regions corresponding to the T3-binding domain and the hinge region of the receptor. These variants alter the ability of the receptor to bind its ligand or the interaction with coactivators and corepressors (3). In addition, mutant receptors interfere with the function of the wild-type TR-β (dominant-negative effect), thus explaining the autosomal dominant inheritance. On the contrary, gross deletions of the *THRβ*, as found in the original family described by Refetoff, follow an autosomal recessive inheritance.

RTHβ is characterised by central hyperthyroidism with raised serum-free T3 (fT3) and serum-free T4 (fT4) associated with non-suppressed levels of TSH, due to impaired sensitivity to thyroid hormone action in the pituitary gland and hypothalamus (3).

Features of thyroid hormone deficiency in TR-β dependent tissues (pituitary gland, hypothalamus, liver and neurosensitive epithelia), as well as thyroid hormone excess in TR-α-dependent tissues (heart, bone, skeletal muscle and brain), may coexist in the same individual.

Thus, the most common clinical manifestations of RTHβ are goiter, sinus tachycardia, liver steatosis and dyslipidaemia, learning disability and hyperactivity disorders, short stature and delayed bone age. However, the clinical picture of RTHβ is highly heterogeneous, since patients can be also asymptomatic, and symptoms may change over time in the same patient (3). Furthermore, patients with the same variant can have different phenotypes, even within the same family, thus suggesting that other genetic and/or epigenetic factors may play a role in determining the RTHβ phenotype (3).

Here, we present the challenging differential diagnosis of a 31-year-old male patient, referred to our unit for a SARS-CoV-2 infection.

## Case presentation

A 31-year-old Caucasian male presented to the Emergency Room of the IRCCS Ospedale Policlinico San Martino (Genoa, Italy) during the first wave of the COVID-19 pandemic with cough, malaise and fever in the context of SARS-CoV-2 infection, for which he had previously started dexamethasone (DMT) treatment at the dose of 6 mg/day.

Due to the presence of atrial fibrillation with rapid ventricular response and a heart rate ranging from 140 to 150 bpm, we started beta-blockers, achieving a heart rate range of 80–90 bpm. Low molecular weight heparin was also added to the corticosteroid therapy, and the patient was admitted to the COVID-19 ward. His past medical history was unremarkable except for a mood disorder treated with valproic acid, while the family history was undetectable as the patient was adopted.

### Investigation

TFTs were measured in the diagnostic workup of atrial fibrillation and revealed a central hyperthyroidism (TSH 4.09 mIU/L, normal range 0.27–4.20; fT3 8.05 pmol/L, normal range 2.76–7.06 and fT4 27.29 pmol/L, normal range 11.97–21.88). Thyroid autoantibodies (thyroid peroxidase antibodies, thyroglobulin antibodies and TSH receptor antibodies) were negative, and the thyroid ultrasound described a normal gland without nodules and normal vascularisation.

Unfortunately, on the third day of hospitalisation, the patient developed dyspnoea and desaturation requiring low-flow oxygen therapy and increasing dose of corticosteroids (methylprednisolone 125 mg/day, equivalent to DMT 23.5 mg), thus impeding a correct differential diagnosis of discrepant TFTs. Therefore, we initiated anti-thyroid therapy with thiamazole 10 mg/day.

On the fifth day of hospitalisation, due to the worsening of heart rate (range: 130–140 bpm), antiarrhythmic treatment with digoxin 0.375 mg/daily was added, and thiamazole increased to 20 mg/day. As expected, TSH further increased following thionamide administration (TSH: 10.07 mIU/L), while circulating thyroid hormones were still increased (fT3: 10.99 pmol/L and fT4: 29.33 pmol/L).

Three days later, the patient showed an improvement in respiratory symptoms, allowing for the weaning of oxygen support and tapering of corticosteroids.

After 18 days, he was finally discharged from the COVID-19 unit with a negative SARS-CoV-2 swab; therefore, additional tests were planned after thiamazole withdrawal for 45 days.

At reassessment, TFTs were still compatible with central hyperthyroidism (TSH: 11.25 mIU/L, fT3: 8.32 pmol/L and fT4: 24.02 pmol/L); SHBG was 23 nmol/L (normal range: 16–64), serum albumin was normal, and the remaining pituitary hormones were within the reference values.

**Figure 1:**
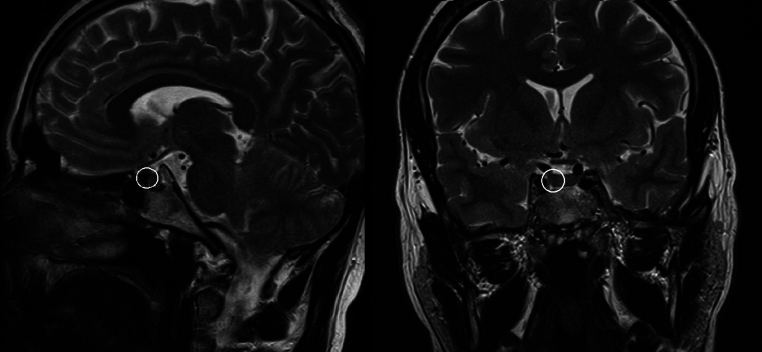
Pituitary MRI T2-weighted coronal and sagittal views where a small area (highlighted) at the right bottom of the gland, consistent with a microadenoma, is observed. The lesion was more evident on T2-weighted sagittal views.

A pituitary MRI revealed a small lesion located at the right bottom of the gland, hypointense in T2-weighted sequences, compatible with a microadenoma (Fig. 1).

As the T3 suppression test was contraindicated due to atrial fibrillation, and TRH not available at that time, an octreotide long-acting release (LAR) test (Table 1) was initiated. In the meantime, genetic testing was performed.

**Table 1:** Octreotide (OCT) LAR test: administration of long-acting release somatostatin analogue (octreotide 30 mg) every 28 days and evaluation of thyroid function values.

Time (day)	OCT LAR injections	TSH (mIU/L; NR: 0.27–4.20)	fT3 (pmol/L; NR: 2.76–7.06)	fT4 (pmol/L; NR: 11.97–21.88)
0*	1°	11.25	8.32	24.02
14	–	13.06	7.65	21.89
28*	2°	8.58	5.48	14.17
42	–	9.94	8.41	23.18
56*	3°	11.76	6.90	25.07
70	–	9.32	6.11	18.79
84	–	10.56	10.73	30.33

*The evaluation of thyroid function tests was performed before octreotide LAR injection.

**Figure 2:**
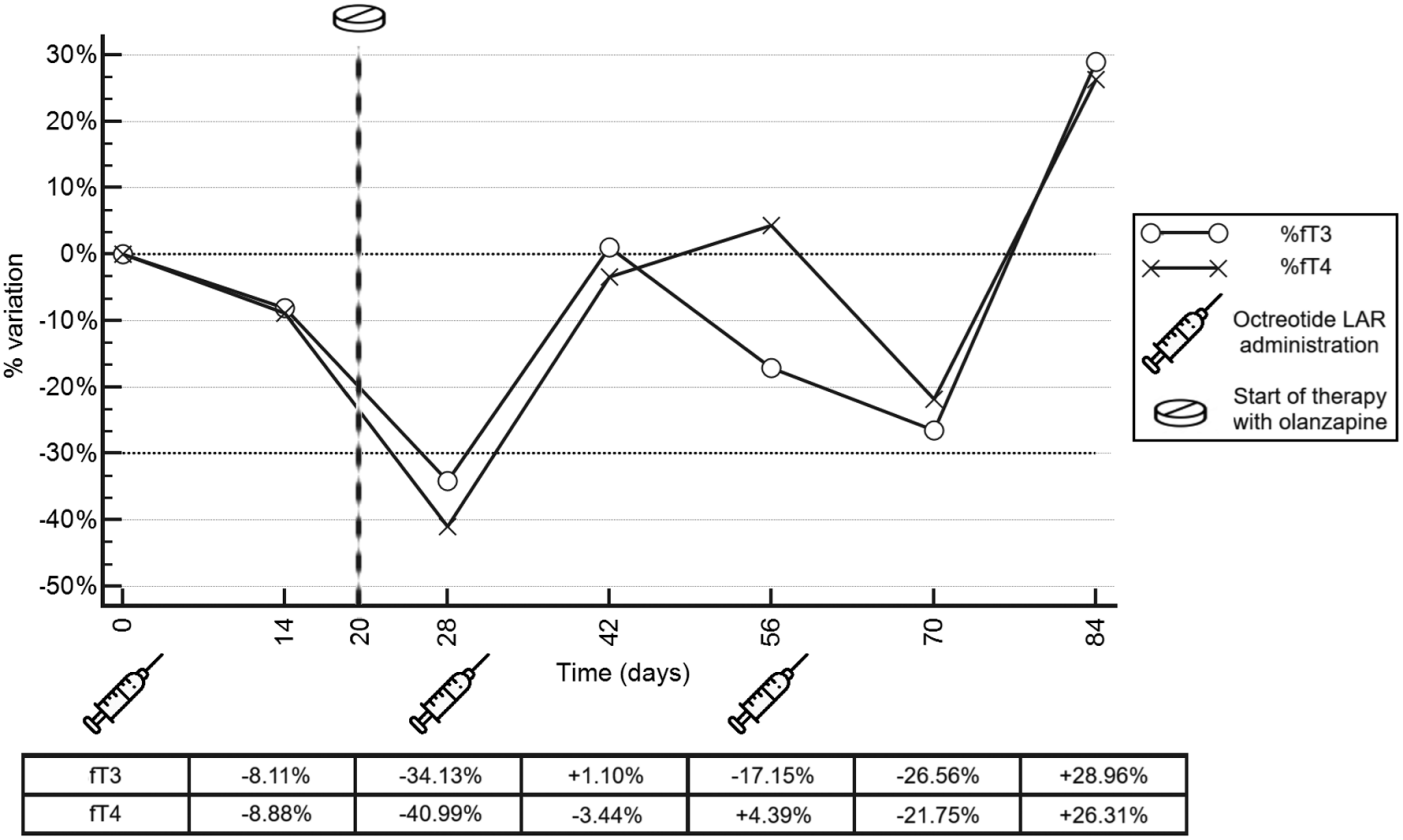
Percentual variation of free thyroid hormones during administration of octreotide LAR. A reduction of levels greater than 30% is consistent with a TSHoma diagnosis.

Interestingly, during the octreotide LAR test, the patient experienced a worsening of his mood disturbances, associated with suicidal ideation requiring hospitalisation and treatment with olanzapine 7.5 mg/day (started at day 20 after the first octreotide LAR injection, Fig. 2).

In the meanwhile, due to the restoration of sinus rhythm with a heart rate range of 60–70 bpm, digoxin and beta-blockers were discontinued without any cardiac upsurge.

The octreotide LAR test was properly conducted, showing a significant decrease in circulating thyroid hormones after the first injection (>30% fT3 and fT4 reduction), suggestive of autonomous TSH secretion by the pituitary gland (TSH-secreting pituitary adenoma, TSHoma). However, the decrease in fT3 and fT4 values was blunted after the second and third octreotide LAR injections, which is unusual for a typical case of TSHoma and more in line with an RTHβ response.

Indeed, the sequencing of the THRβ gene revealed a heterozygous variant in codon 453, with a substitution of cytosine for thymine at position 1357, which determines a substitution of proline for serine at position 453 of THRβ (Pro453Ser). Therefore, the diagnosis of RTHβ was established. Accordingly, medical treatment with triiodothyroacetic acid (TRIAC) was initiated.

### Outcome and follow-up

Two years from the first hospitalisation, the patient has normal thyroid function on TRIAC at a dosage of 1050 mg/day (TSH: 2.75 mIU/L, fT3: 5.76 pmol/L and fT4: 15.77 pmol/L). No further psychiatric interventions have been needed, the small pituitary lesion remained stable at the MRI, and the patient continues regular follow-up at our outpatient clinic.

## Discussion

Central hyperthyroidism is a rare clinical entity encompassing RTHβ and TSHoma. The differential diagnosis of central hyperthyroidism requires proper laboratory, radiological and genetic investigations. In our case, the challenge was made more compelling due to the concomitant SARS-CoV-2 infection, which required treatments that may interfere with TFT assessment, including steroids and heparin.

Nevertheless, the co-occurrence of discrepant TFTs and cardiac arrhythmias was highly suggestive of a genuine central hyperthyroidism. At that time, most of the healthcare facilities were converted into COVID-19 units to face the first wave of the pandemic, and the differential diagnosis was deferred. We administered a short course of thionamides to control hyperthyroidism, and treatment was modulated to avoid excessive increases in TSH, which may cause pituitary hyperplasia in RTHβ or tumour growth in TSHomas with chronic administration.

The past medical history of our patient was negative for any cardiac rhythm alteration, but we observed atrial fibrillation at hospital admission. This is not surprising, since RTHβ patients may develop major cardiovascular events earlier than the general population. In addition, the new onset of atrial fibrillation is a possible complication of COVID-19, with an estimated incidence in patients without a pre-existing cardiac history of 4% (4). Furthermore, treatment with corticosteroids started before hospitalisation because of SARS-CoV-2 infection, may have triggered atrial fibrillation (5). In support of these hypotheses, when corticosteroid therapy was increased because of hypoxaemia, the heart rhythm worsened, and a modulation of antiarrhythmic therapy was needed.

In our case, the diagnostic steps carried out to differentiate RTHβ and a TSHoma started with an evaluation of the other anterior pituitary hormones than TSH, which resulted within the normal range, and SHBG dosage. SHBG is a marker of peripheral thyroid hormone action, and the finding of a normal value is usually more in line with the diagnosis of RTHβ. Interestingly, a contrast-enhanced MRI of the pituitary region revealed a small lesion compatible with a microadenoma. The MRI, because of its high reproducibility and the relatively simple execution, is considered a necessary step during the diagnostic workup, but it could be misleading because of its low specificity. Indeed, a pituitary lesion may be found in up to 24% of patients with RTHβ (6), as well as in a similar percentage of the general population.

Another important factor that can help to differentiate RTHβ from TSHoma is the family history: to our knowledge, familial cases of TSHoma have been described only in two families with multiple endocrine neoplasia type 1 or familial isolated pituitary adenoma due to AIP variants (7). As reported, our patient was adopted, and it was not possible to obtain a family history.

The pituitary finding, although small in size and not specific, led us to perform additional testing. Despite TSHomas being more likely to be macroadenomas, up to 30% could present as microadenomas.

The T3 suppression test is the most specific, as TSH suppression has never been reported in TSHomas, but it is contraindicated in cases of atrial fibrillation. For this reason, we opted for a test with octreotide LAR. This test consists of the chronic administration of a long-acting somatostatin analogue every 28 days for at least two injections with a standardised assessment of TFTs at baseline and day 56 (8). The administration of long-acting octreotide normalises or at least causes a reduction greater than 30% of fT3 and fT4 in most of the patients with a TSHoma, while RTHβ patients do not show any response (7, 8). Moreover, somatostatin analogues such as octreotide and lanreotide are effective treatments for TSHoma, as recommended by guidelines when pituitary surgery is contraindicated or declined (9), and even suggested as first-line therapy by some authors (10).

In our patient, we investigated thyroid function at baseline and every 14 days during the administration of octreotide LAR 30 mg (2). The initial reduction of fT3 and fT4 (34% and 40%, respectively) observed on day 28 was suspicious of a TSHoma; however, during chronic administration, thyroid hormone increased again, as rarely found in RTHβ (8, 11).

Antipsychotic treatment may have contributed to the marked reduction of fT3 and fT4 observed after the first octreotide injection: indeed, antipsychotic drugs, particularly quetiapine and olanzapine, are associated with lower fT4 levels, and this association is more pronounced if combined with other psychotropic drugs (12). However, this effect should have persisted over time (olanzapine treatment was continued during the whole test), but this was not the case in our patient.

The sequencing of the TRHβ gene showed a single nucleotide variant causing a substitution of proline for serine at position 453 of THRß (Pro453Ser), which led us to the diagnosis of RTHβ.

Codon 453, placed in Cluster 1 of the three hotspots, is a common site where variants occur, including eight different substitutions (P453T, S, A, N, Y, H and R), and a total of 74 families described as harbouring mutations of this site. The variant is located in the T3-binding domain and exhibits diminished affinity to its ligand, an impaired interaction with co-regulators, as well as a domain negative effect over the wild-type receptor (13).

Since the diagnosis, the patient underwent medical treatment with TRIAC, a T3 analogue with a demonstrated higher affinity to TRβ1 than T3 (14). The treatment was started with the objective of maintaining an acceptable balance between over-stimulation of predominantly TRα-expressing tissues, such as the heart and brain, and under-stimulation of predominantly TRβ-expressing tissues, such as the pituitary gland and liver (15). Several *in vitro* and *in vivo* studies demonstrate that TRIAC reduces basal and TRH-induced serum TSH levels, resulting in a subsequent decrease of serum TH levels, especially in variants that fall within Clusters 1 and 2 of the three hotspots of the THRβ gene (16).

Few cases of RTHβ associated with a TSHoma, confirm the extremely rare concomitant presence of RTHβ and a TSHoma (17). In our case, this rare occurrence is excluded by the changes in TFTs observed during TRIAC administration, which were not compatible with the secretion of TSH refractory to the negative feedback (Table 1 and Fig. 2) found in TSHoma. Additionally, the small pituitary lesion remained stable during follow-up, and the patient did not undergo neurosurgery. The TRIAC therapy guaranteed clinical and biochemical stability, and the patient continued regular follow-up at our outpatient clinic.

In conclusion, we have reported the case of a patient with elevated thyroid hormones and normal/slightly elevated TSH due to RTHβ (Pro453Ser variant), with a clinical presentation of atrial fibrillation, during SARS-CoV-2 infection.

The concomitant finding of a small pituitary lesion, the inability to obtain a family history or to test the relatives of the patient, and the presence of a psychiatric disorder treated with antipsychotic drugs made the diagnosis even more challenging.

Finally, the results of the octreotide LAR test were also difficult to interpret, since circulating thyroid hormones (fT3 and fT4) markedly reduced after the first injection, although a lack of response was observed following the two additional injections (resembling a tachyphylaxis phenomenon).

This unusual response to octreotide LAR administrations highlights the need for careful and proper conduct of this test. An early discontinuation could lead to a biochemical response suggestive of TSHoma (false positive result).

In the differential diagnosis between RTHβ and TSHoma, an appropriate level of clinical suspicion associated with careful evaluation of potential interfering factors, such as concomitant treatments during tests, is necessary to avoid unnecessary and potentially harmful therapies.

## Declaration of interest

DF has either received honoraria for lectures from or is on advisory boards of Recordati, Ipsen and Novartis-AAA. DF has also received research grants from Camurus and Pfizer. DF serves on the Executive Committee of the Italian Endocrine Society (SIE). FG has received personal honoraria for lectures, manuscript writing, educational events and consultancy from Ipsen, Novartis, Pfizer and Recordati. FG serves on the Executive Committee of the European Neuroendocrine Association (ENEA).

## Funding

This case report did not receive any specific grant from any funding agency in the public, commercial or not-for-profit sector.

## Patient consent

The patient agreed to this publication. A written informed consent for the publication of clinical details was obtained from the patient.

## Author contribution statement

Evaluation and clinical decision, SG, FC and FG; writing – original draft preparation, CP and SB; writing – review and editing, CP, SG, LV and FG; supervision, IC, LP and DF; funding acquisition, DF.

## References

[1] Refetoff S Bassett JH Beck-Peccoz P Bernal J Brent G Chatterjee K De Groot LJ Dumitrescu AM Jameson JL Kopp PA, *et al.* Classification and proposed nomenclature for inherited defects of thyroid hormone action, cell transport, and metabolism. Journal of Clinical Endocrinology and Metabolism 2014 99 768–770. (10.1210/jc.2013-3393) 24823702 PMC3942236

[2] Refetoff S DeWind LT & DeGroot LJ. Familial syndrome combining deaf-mutism, stuppled epiphyses, goiter and abnormally high PBI: possible target organ refractoriness to thyroid hormone. Journal of Clinical Endocrinology and Metabolism 1967 27 279–294. (10.1210/jcem-27-2-279) 4163616

[3] Pappa T & Refetoff S. Resistance to thyroid hormone beta: a focused review. Frontiers in Endocrinology 2021 12 656551. (10.3389/fendo.2021.656551) 33868182 PMC8044682

[4] Inciardi RM Adamo M Lupi L Cani DS Di Pasquale M Tomasoni D Italia L Zaccone G Tedino C Fabbricatore D, *et al.* Characteristics and outcomes of patients hospitalized for COVID-19 and cardiac disease in Northern Italy. European Heart Journal 2020 41 1821–1829. (10.1093/eurheartj/ehaa388) 32383763 PMC7239204

[5] Christiansen CF Christensen S Mehnert F Cummings SR Chapurlat RD & Sørensen HT. Glucocorticoid use and risk of atrial fibrillation or flutter: a population-based, case-control study. Archives of Internal Medicine 2009 169 1677–1683. (10.1001/archinternmed.2009.297) 19822824

[6] Campi I Covelli D Moran C Fugazzola L Cacciatore C Orlandi F Gallone G Chatterjee K Beck-Peccoz P & Persani L. The differential diagnosis of discrepant thyroid function tests: insistent pitfalls and updated flow-chart based on a long-standing experience. Frontiers in Endocrinology 2020 11 432. (10.3389/fendo.2020.00432) 32733382 PMC7358450

[7] Beck-Peccoz P Persani L Mannavola D & Campi I. Pituitary tumours: TSH-secreting adenomas. Best Practice and Research. Clinical Endocrinology and Metabolism 2009 23 597–606. (10.1016/j.beem.2009.05.006) 19945025

[8] Mannavola D Persani L Vannucchi G Zanardelli M Fugazzola L Verga U Facchetti M & Beck-Peccoz P. Different responses to chronic somatostatin analogues in patients with central hyperthyroidism. Clinical Endocrinology 2005 62 176–181. (10.1111/j.1365-2265.2004.02192.x) 15670193

[9] Beck-Peccoz P Lania A Beckers A Chatterjee K & Wemeau JL. 2013 European Thyroid Association guidelines for the diagnosis and treatment of thyrotropin-secreting pituitary tumors. European Thyroid Journal 2013 2 76–82. (10.1159/000351007) 24783044 PMC3821512

[10] Gatto F Grasso LF Nazzari E Cuny T Anania P Di Somma C Colao A Zona G Weryha G Pivonello R, *et al.* Clinical outcome and evidence of high rate post-surgical anterior hypopituitarism in a cohort of TSH-secreting adenoma patients: might somatostatin analogs have a role as first-line therapy? Pituitary 2015 18 583–591. (10.1007/s11102-014-0611-8) 25326851

[11] Beck-Peccoz P Mariotti S Guillausseau PJ Medri G Piscitelli G Bertoli A Barbarino A Rondena M Chanson P & Pinchera A. Treatment of hyperthyroidism due to inappropriate secretion of thyrotropin with the somatostatin analog SMS 201–995. Journal of Clinical Endocrinology and Metabolism 1989 68 208–214. (10.1210/jcem-68-1-208) 2491862

[12] Vedal TSJ Steen NE Birkeland KI Dieset I Reponen EJ Laskemoen JF Rødevand L Melle I Andreassen OA Molden E, *et al.* Free thyroxine and thyroid-stimulating hormone in severe mental disorders: a naturalistic study with focus on antipsychotic medication. Journal of Psychiatric Research 2018 106 74–81. (10.1016/j.jpsychires.2018.09.014) 30292780

[13] Refetoff S Weiss RE Wing JR Sarne D Chyna B & Hayashi Y. Resistance to thyroid hormone in subjects from two unrelated families is associated with a point mutation in the thyroid hormone receptor beta gene resulting in the replacement of the normal proline 453 with serine. Thyroid 1994 4 249–254. (10.1089/thy.1994.4.249) 7833659

[14] Takeda T Suzuki S Liu RT & DeGroot LJ. Triiodothyroacetic acid has unique potential for therapy of resistance to thyroid hormone. Journal of Clinical Endocrinology and Metabolism 1995 80 2033–2040. (10.1210/jcem.80.7.7608251) 7608251

[15] Groeneweg S Peeters RP Visser TJ & Visser WE. Therapeutic applications of thyroid hormone analogues in resistance to thyroid hormone (RTH) syndromes. Molecular and Cellular Endocrinology 2017 458 82–90. (10.1016/j.mce.2017.02.029) 28235578

[16] Anzai R Adachi M Sho N Muroya K Asakura Y & Onigata K. Long-term 3,5,3'-triiodothyroacetic acid therapy in a child with hyperthyroidism caused by thyroid hormone resistance: pharmacological study and therapeutic recommendations. Thyroid 2012 22 1069–1075. (10.1089/thy.2011.0450) 22947347

[17] Teng X Jin T Brent GA Wu A Teng W & Shan Z. A patient with a thyrotropin-secreting microadenoma and resistance to thyroid hormone (P453T). Journal of Clinical Endocrinology and Metabolism 2015 100 2511–2514. (10.1210/jc.2014-3994) 25867808 PMC5393528

